# Hyperuricemia as a prognostic marker for long-term outcomes in patients with myocardial infarction with nonobstructive coronary arteries

**DOI:** 10.1186/s12986-021-00636-2

**Published:** 2021-12-20

**Authors:** Wenjian Ma, Side Gao, Sizhuang Huang, Jiansong Yuan, Mengyue Yu

**Affiliations:** 1 Department of Cardiology, Xinhua Hospital, Shanghai Jiaotong University School of Medicine, Shanghai, China; 2 Department of Cardiology, Fuwai Hospital, National Center for Cardiovascular Diseases, Chinese Academy of Medical Sciences and Peking Union Medical College, Bei Li Shi Rd 167, Beijing, 100037 PR China

**Keywords:** Myocardial infarction with nonobstructive coronary arteries (MINOCA), Hyperuricemia, Cardiovascular outcomes

## Abstract

**Background:**

Hyperuricemia (HUA) has been proved as a predictor of worse outcomes in patients with coronary artery disease. Here, we investigated the prognostic value of HUA in a distinct population with myocardial infarction with nonobstructive coronary arteries (MINOCA).

**Methods:**

A total of 1179 MINOCA patients were enrolled and divided into HUA and non-HUA groups. HUA was defined as a serum uric acid level ≥ 420 μmol/L in men or ≥ 357 μmol/L in women. The primary study endpoint was a composite of major adverse cardiovascular events (MACE), including all-cause death, nonfatal MI, nonfatal stroke, revascularization, and hospitalization for unstable angina or heart failure. Kaplan–Meier, Cox regression, and receiver-operating characteristic analyses were performed.

**Results:**

Patients with HUA (prevalence of 23.5%) had a significantly higher incidence of MACE (18.7% vs. 12.8%; *p* = 0.015) than patients without during the median follow-up of 41.7 months. HUA was closely associated with an increased risk of MACE even after multivariable adjustment (hazard ratio 1.498, 95% confidence interval: 1.080 to 2.077; *p* = 0.016). HUA remained a robust risk factor of MACE after propensity score matching analysis. Moreover, HUA showed an area under the curve (AUC) of 0.59 for predicting MACE. Incorporation of HUA to the thrombolysis in myocardial infarction (TIMI) score yielded a significant improvement in discrimination for MACE.

**Conclusions:**

HUA was independently associated with poor prognosis after MINOCA. Routine assessment of HUA may facilitate risk stratification in this specific population.

**Supplementary Information:**

The online version contains supplementary material available at 10.1186/s12986-021-00636-2.

## Introduction

Acute myocardial infarction (AMI) remains a major cause of morbidity and mortality of cardiovascular diseases (CVD) worldwide [1], and recently, a distinct population with myocardial infarction with nonobstructive coronary arteries (MINOCA) has been increasingly recognized due to the widespread use of coronary angiography. Among all AMIs, patients with MINOCA account for 5% to 10% and they are younger and more often women compared to those with AMI and obstructive coronary artery disease [2–5]. Several studies have showed that the prognosis of MINOCA is not necessarily benign and patients with MINOCA are still at considerable risks for long-term cardiovascular (CV) events despite the optimal medical therapies [6–9]. Therefore, it is of necessity to find potential residual risk factors and improve prognosis for MINOCA population.

Serum uric acid (UA) is the final product of purine metabolism catalyzed by xanthine oxidase [10]. Elevated UA levels are closely linked with hypertension, diabetes, obesity, metabolic syndrome, and kidney diseases [11–14]. More importantly, emerging evidence indicates that hyperuricemia (HUA) is commonly seen and is an independent predictor of mortality and adverse CV events in general population [15–19]. Recent studies further verified the prognostic value of HUA in various subgroups with coronary artery disease (CAD), including stable CAD, acute coronary syndrome (ACS), and those undergoing percutaneous coronary intervention (PCI) [20–26], whereas some studies failed to confirm this association [27, 28]. Despite these data, few of them have addressed the implications of HUA in MINOCA, and the impact of HUA on long-term outcomes after MINOCA remains unclear. Here, we explored the prognostic value of HUA in MINOCA patients, and investigated whether it could improve risk stratification in this specific population.

## Methods

### Study population

**Fig. 1:**
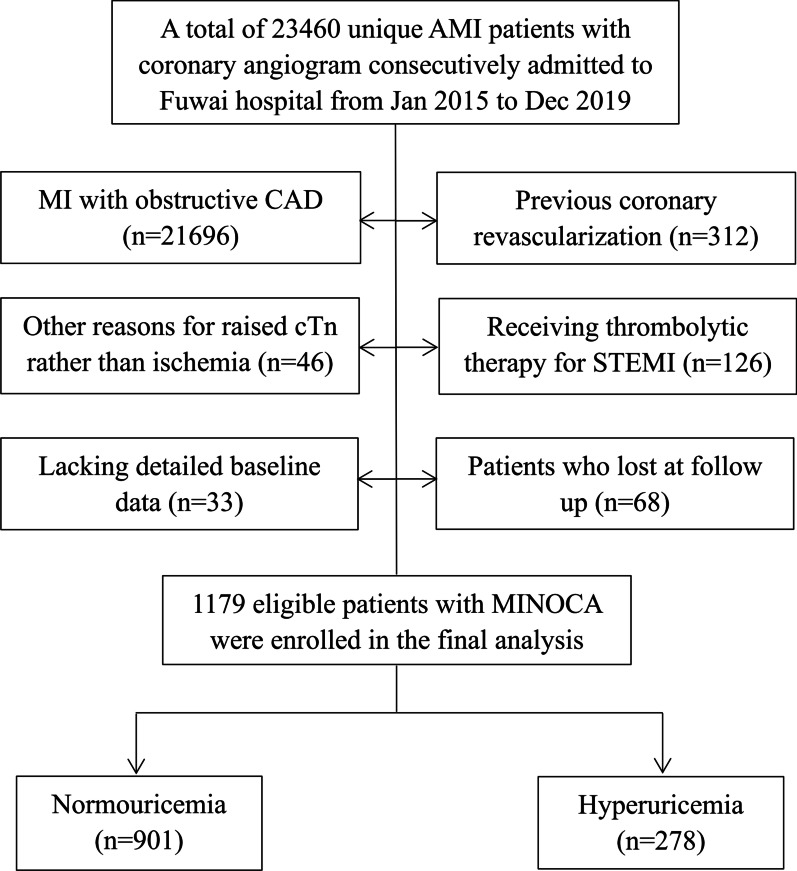
Study flowchart

This was a single-center, prospective and observational cohort study of patients with MINOCA. From January 2015 to December 2019, a total of 23,460 unique AMI patients with coronary angiogram were consecutively hospitalized in Fuwai hospital, including non ST-segment elevation myocardial infarction (NSTEMI) and ST-segment elevation myocardial infarction (STEMI). Patients were diagnosed with MINOCA if they met the 4^th^ universal definition of AMI [29] and the coronary angiography did not show a stenosis of ≥ 50% in epicardial coronary arteries [2]. Patients were excluded due to: (1) presence of obstructive CAD (n = 21,696); (2) prior revascularization (n = 312); (3) thrombolytic therapy for STEMI since the coronary lesion may be affected by thrombolysis (n = 126); (4) alternate explanations for elevated troponin rather than coronary-related causes (e.g., acute heart failure, myocarditis, pulmonary embolism, takotsubo syndrome, n = 46); (5) lack of detailed baseline data (n = 33); (6) lost at follow up (n = 68). As a result, 1179 eligible MINOCA patients were enrolled into the final analysis (Fig. 1). Patients were prescribed the evidence-based secondary therapies, including dual anti-platelet therapy (DAPT), statins, β-blocker, and angiotensin-converting enzyme inhibitor (ACEI) or angiotensin receptor antagonist (ARB) [30, 31]. This study was approved by the Ethics Committee of Fuwai hospital and complied with the Declaration of Helsinki. All enrolled subjects provided the written informed consent.

### Data collection

Patients’ baseline data were collected and verified from medical records. The blood samples were routinely collected from cubital vein at fasting conditions for biochemical measurement during first 48 h since admission. The plasma concentrations of uric acid (UA), fasting blood glucose (FBG), triglyceride (TG), total cholesterol (TC), low-density lipoprotein cholesterol (LDL-C), high-density lipoprotein cholesterol (HDL-C), creatinine, and high-sensitive C-reactive protein (hs-CRP) were tested via an automatic biochemistry analyzer. Specifically, UA was analyzed with an automated biochemical analyzer (Hitachi 7150, Tokyo, Japan) in an enzymatic assay using a UA commercial kit (uricase-peroxidase method). A validated standard of UA was used for calibration, and the coefficient of variation of repetitive measurements was < 10%. The N-terminal po-B-type natriuretic peptide (NT-proBNP) at admission and peak cardiac troponin I (TnI) values were recorded. The left ventricular ejection fraction (LVEF) was measured by echocardiography using biplane Simpson method. The Thrombolysis in Myocardial Infarction (TIMI) score was calculated since admission as previously described [32, 33].

### Definitions and outcomes

In line with previous studies, hyperuricemia (HUA) was defined as a serum UA level ≥ 420 μmol/L (7.0 mg/dL) in males and ≥ 357 μmol/L (6.0 mg/dL) in females [10]. Diabetes mellites (DM) was defined with FBG ≥ 7.0 mmol/L, 2-h plasma glucose ≥ 11.1 mmol/L, or having known DM. Hypertension was defined as repeated blood pressure ≥ 140/90 mmHg or use of anti-hypertensive drugs. Dyslipidemia was diagnosed by past history or having LDL-C ≥ 3.4 mmol/L, HDL-C < 1.0 mmol/L, or TG ≥ 1.7 mmol/L.

The primary study endpoint was a composite of major adverse cardiovascular events (MACE), including all-cause death, nonfatal MI, revascularization, nonfatal stroke, and hospitalization for unstable angina (UA) or heart failure (HF). The MACE was assessed as time to first event. The secondary endpoints included each component of MACE and the composite “hard” endpoint of death, nonfatal MI, revascularization, and nonfatal stroke. Reinfarction was diagnosed according to the 4^th^ universal definition of MI [29]. Revascularization was performed at the operator’s discretion due to recurrent ischemia and progression of coronary lesion. Stroke was defined by the presence of neurological dysfunction and vascular brain injury caused by cerebral ischemia or hemorrhage [34]. Hospitalization for UA or HF reflected the clinical status and quality of life after AMI. Patients were regularly and actively followed up at clinics or through telephone contact at 6 month intervals by a team of independent well-trained researchers. The endpoints were confirmed by at least two professional cardiologists.

### Statistical analysis

Data were expressed as mean ± standard deviation (SD) or median with interquartile range for continuous variables and numbers with percentages for categorical variables. Differences were assessed using Student’s t test or Mann–Whitney U test for continuous variables and Pearson’s χ^2^ or Fisher’s exact test for categorical variables. Cumulative hazard ratio of MACE between HUA and non-HUA groups were showed by Kaplan–Meier analysis and compared using the log-rank test. The univariable and multivariable Cox proportional regression analyses were used to identify association of HUA with CV outcomes. The risk of MACE was adjusted by multiple clinically relevant variables, including age, sex, BMI, MI classification (NSTEM or STEMI), hypertension, diabetes and dyslipidemia. Hazard ratio (HR) with 95% confidence interval (CI) were calculated. The effect of HUA on MACE risk was further assessed by propensity score matching (PSM) and sex subgroup analyses, in which detailed explanations were shown in the supplementary data. Discrimination was defined with areas under the curve (AUC) using a receiver-operating characteristic curve (ROC) analysis. The AUC values were classified as small (0.56–0.63), moderate (0.64–0.70) or strong (≥ 0.71) [35] and compared by Delong’s test [36]. To evaluate the incremental predictive value of HUA for MACE, a combined risk model was generated by incorporating HUA into the original TIMI risk score based on multivariate Cox model. A two-sided analysis with a P value < 0.05 was considered statistically significant. Data were analyzed using SPSS V.22.0 (SPSS Inc., Chicago, USA) and R language V.3.6.3 (Feather Spray).

## Results

### Baseline characteristics

Patients were divided into normouricemia group and HUA group. The incidence of HUA was 23.5% in all MINOCA patients (Fig. 1). The plasma UA concentrations were normally distributed in the population (Additional file 1: Fig. S1). As shown in Table 1, patients with HUA were younger and more often male. They had higher BMI, higher percent of STEMI, higher TG and lower HDL-C. There were no significant differences in the rate of hypertension, diabetes, dyslipidemia, prior MI and in-hospital medication. Also, the Killip class, LVEF level, TIMI risk score, and values of FBG, TC, LDL-C, creatinine, hs-CRP, NT-proBNP, and peak TnI were similar between the groups. In this regard, despite the differences in demographics, the comorbidities and general CV risk profiles were comparable in patients with or without HUA.

**Table.1:** Baseline characteristics and clinical outcomes in MINOCA patients with or without hyperuricemia

Variable	Total (n = 1179)	Normouricemia (n = 901)	Hyperuricemia (n = 278)	*p* value
Male, n (%)	867 (73.5%)	642 (71.2%)	225 (80.9%)	0.001
Age, years	55.7 ± 11.8	56.6 ± 11.1	53.0 ± 13.5	< 0.001
BMI, kg/m^2^	25.4 ± 3.7	25.1 ± 3.5	26.5 ± 4.3	< 0.001
STEMI, n (%)	475 (40.2%)	344 (38.1%)	131 (47.1%)	0.008
Emergent angiography, n (%)	159 (13.4%)	125 (13.8%)	34 (12.2%)	0.483
Past history				
Hypertension	630 (53.4%)	474 (52.6%)	156 (56.1%)	0.305
Diabetes	187 (15.8%)	151 (16.7%)	36 (12.9%)	0.129
Dyslipidemia	686 (58.1%)	512 (56.8%)	174 (62.5%)	0.089
Previous MI	58 (4.9%)	44 (4.8%)	14 (5.0%)	0.918
Killip class ≥ 2, n (%)	89 (7.5%)	64 (7.1%)	25 (8.9%)	0.514
LVEF, %	60.5 ± 7.5	60.8 ± 6.7	60.3 ± 8.4	0.112
TIMI risk score	3.4 ± 1.3	3.3 ± 1.2	3.5 ± 1.3	0.076
Blood test				
Uric acid, μmol/L	343.4 ± 94.2	305.1 ± 65.1	467.9 ± 59.9	< 0.001
FBG, mmol/L	5.69 ± 1.68	5.72 ± 1.72	5.66 ± 1.63	0.630
TG, mmol/L	1.44 (1.05, 2.00)	1.36 (1.03, 1.93)	1.67 (1.28, 2.29)	< 0.001
TC, mmol/L	3.92 ± 0.87	3.91 ± 1.01	3.92 ± 0.91	0.760
LDL-C, mmol/L	2.29 ± 0.76	2.29 ± 0.73	2.29 ± 0.83	0.971
HDL-C, mmol/L	1.08 ± 0.29	1.11 ± 0.30	0.98 ± 0.24	0.001
Creatinine, μmol/L	83.13 ± 15.89	82.92 ± 14.90	84.23 ± 17.03	0.202
hs-CRP, mg/L	2.20 (1.03, 5.75)	2.14 (0.96, 5.80)	2.37 (1.25, 5.69)	0.128
NT-proBNP, pg/mL	372 (112, 683)	368 (108, 672)	379 (125, 694)	0.132
Peak TnI, ng/mL	3.24 (0.72, 6.51)	3.11 (0.65, 6.41)	3.35 (0.98, 6.93)	0.157
In-hospital medication				
DAPT	1091 (92.5%)	837 (92.8%)	254 (91.3%)	0.396
Statin	1130 (95.8%)	861 (95.5%)	269 (96.7%)	0.380
Beta-blocker	860 (72.9%)	655 (72.6%)	205 (73.7%)	0.732
ACEI or ARB	759 (64.3%)	572 (63.4%)	187 (67.2%)	0.103
CV outcomes				
MACE	168 (14.2%)	116 (12.8%)	52 (18.7%)	0.015
Death, nonfatal MI, stroke or revascularization	102 (8.6%)	69 (7.6%)	33 (11.8%)	0.029
All-cause death	18 (1.5%)	11 (1.2%)	7 (2.5%)	0.123
Nonfatal MI	41 (3.4%)	27 (2.9%)	14 (5.0%)	0.105
Revascularization	46 (3.9%)	33 (3.6%)	13 (4.6%)	0.445
Nonfatal stroke	12 (1.0%)	7 (0.7%)	5 (1.7%)	0.138
Hospitalization for UA	71 (6.0%)	56 (6.2%)	15 (5.3%)	0.616
Hospitalization for HF	48 (4.0%)	27 (2.9%)	21 (7.5%)	0.001

Hyperuricemia was defined as a serum uric acid level ≥ 420 μmol/ L in males and ≥ 357 μmol/L in females. BMI: body mass index, STEMI: ST-segment elevation myocardial infarction, LVEF: left ventricular ejection fraction, TIMI: Thrombolysis in Myocardial Infarction, FBG: fasting blood glucose, TG: triglyceride, TC: total cholesterol, LDL-C: low-density lipoprotein cholesterol, HDL-C: high-density lipoprotein cholesterol, hs-CRP: high-sensitive C-reactive protein, NT-proBNP: N-terminal pro-B-type natriuretic peptide, TnI: Troponin I, DAPT: dual anti-platelet therapy, ACEI: angiotensin-converting enzyme inhibitor, ARB: angiotensin receptor antagonist, MACE: major adverse cardiovascular events, UA: unstable angina, HF: heart failure

### Association between HUA and outcomes

**Fig. 2:**
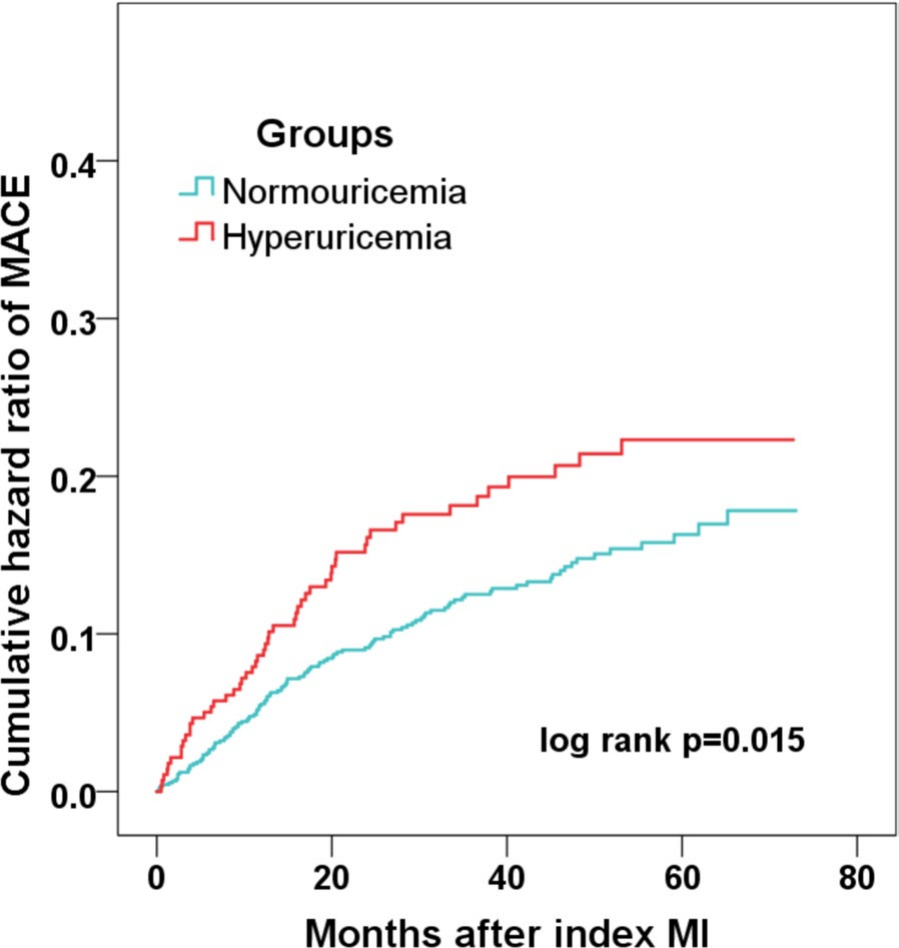
Incidence of MACE in MINOCA patients with or without hyperuricemia. Kaplan–Meier analysis showing the cumulative hazard ratio of MACE in patients with MINOCA with or without hyperuricemia

During the median follow-up of 41.7 months, 168 patients developed MACE (18 died, 41 had recurrent MI, 46 underwent revascularization, 12 suffered stroke, 71 was hospitalized for UA and 48 hospitalized for HF) (Table 1). The profiles in patients with or without MACE were also compared and those who developed MACE had more UA levels (Additional file 1: Table S1). Consistently, patients with HUA had a significantly higher incidence of MACE than patient without (18.7% vs. 12.8%; *p* = 0.015) (Table 1). The rate of the composite “hard” endpoint of death, recurrent MI, revascularization or stroke also increased in HUA group (11.8% vs. 7.6%; *p* = 0.029). In addition, the Kaplan–Meier analysis showed that the cumulative incidence of MACE was significantly higher in patients with HUA (log rank *p* = 0.015) (Fig. 2). As for each component of MACE, the incidence of death, MI, stroke or revascularization were slightly higher in HUA group, but no significant differences were observed. Interestingly, patients with HUA had a significantly higher rate of HF (Table 1).

At multivariate Cox analysis, the HUA was significantly associated with an increased risk of MACE after multivariable adjustment (HR 1.498, 95% CI: 1.080–2.077, *p* = 0.016) (Table 2). As a continuous variable, the UA levels also correlated with the adjusted risk of MACE (for per 1SD increase in UA, HR 1.012, 95% CI: 1.006–1.018, *p* = 0.005). We further analyzed the potential risk factors and HUA remained an independent predictor of MACE (Additional file 1: Table S2). This association was still significant in the 273 matched pairs of MINOCA patients with or without HUA after PSM (Additional file 1: Table S3), indicating that HUA may be a residual risk factor for MINOCA patients. The UA levels and CV outcomes were also compared between sexes. Men had higher UA than women while no sex gaps in prognosis were observed (Additional file 1: Table S4).

**Table.2:** Association between uric acid levels and the risk of MACE

Group	Unadjusted Cox analysis		Adjusted Cox analysis	
	HR (95% CI)	P value	HR (95% CI)	P value
Serum UA, per 1SD increase	1.014 (1.007–1.020)	0.001	1.012 (1.006–1.018)	0.005
Normouricemia	Reference		Reference	
Hyperuricemia	1.663 (1.188–2.327)	0.003	1.498 (1.080–2.077)	0.016

Hazard ratio (HR) was adjusted by age, sex, BMI, MI type (STEMI or NSTEMI), hypertension, diabetes and dyslipidemia in multivariate Cox analysis. HR: hazard ratio, CI: confidence interval, SD: standard deviation, UA: uric acid

### Predictive value of HUA for MACE

**Fig. 3:**
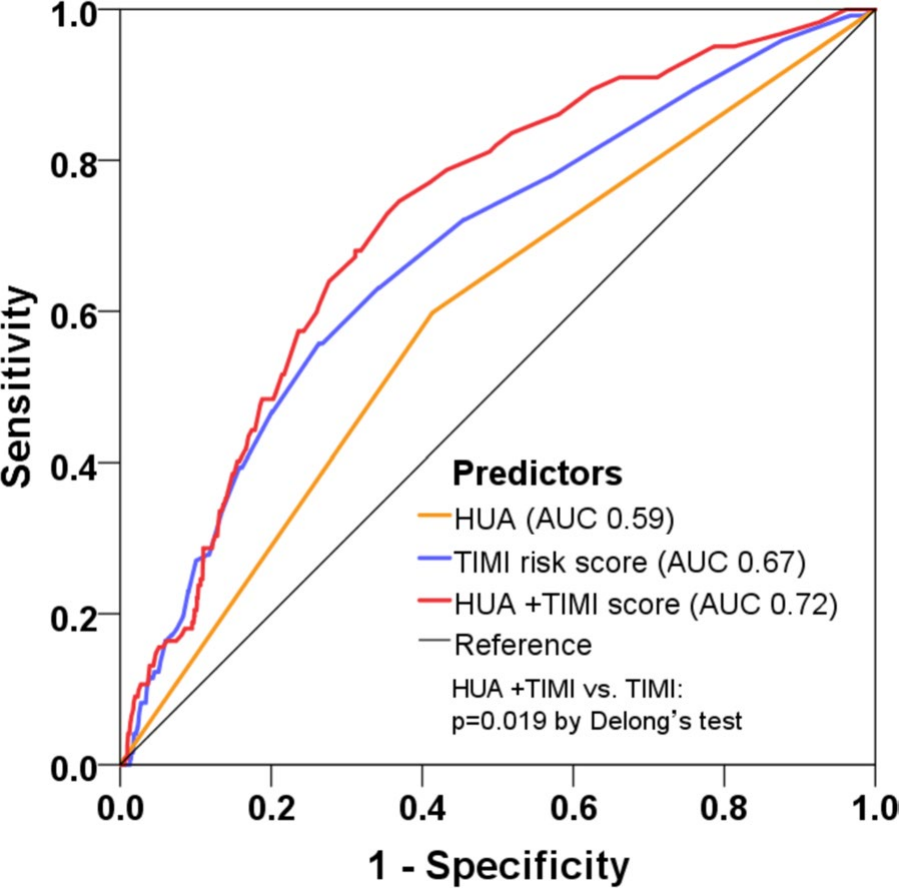
Model improvement in predicting MACE. Receiver operating characteristic curves showing the predictive value of hyperuricemia (HUA), TIMI risk score, and the combined model incorporating HUA and TIMI score. TIMI: Thrombolysis in Myocardial Infarction, AUC: area under the curve

The ROC analysis showed the discriminatory ability of HUA (AUC 0.59) and TIMI risk score (AUC 0.67) for MACE prediction (Fig. 3). When adding HUA to the original TIMI score, the combined model enabled a more accurate prediction of MACE (AUC 0.72) and accordingly yielded a significant improvement in risk prediction (ΔAUC 0.05, *p* = 0.019 by DeLong’s test).

## Discussion

In the present study, we found that HUA was frequently seen and was independently associated with worse prognosis in patients with MINOCA. Moreover, HUA improved the outcome prediction when added to a well-established risk score. These data indicate that assessment of serum UA levels may provide valuable prognostic information, and further support the utility of HUA for risk stratification in the contemporary real-world management of MINOCA.

MINOCA represents a distinct clinical entity with multiple underlying mechanisms, including plaque rupture, erosion, thromboembolism, coronary spasm, dissection, and myocardial supply/demand mismatch. Some non-ischemic diseases such as myocarditis may also mimic the presentation of MINOCA [5]. More recently, the term MINOCA has been used to primarily describe patients with coronary-related ischemia. We used this criteria and established a long-term cohort of MINOCA with a relatively large sample size. The prevalence of MINOCA is 5.1% in our study, which is close to the estimated 6% in all AMIs [4]. As reported, patients with MINOCA were more likely to be younger, female, and had fewer comorbidities. Nearly one-third of MINOCA would present with STEMI [4]. We described the clinical profiles of MINOCA as well. Also, we found that the prognosis of MINOCA was not a trivial thing. During the median follow-up of 3.5 years, 1.5% of MINOCA patients died and 14.2% of them developed MACE. Similarly, previous studies showed that these patients were still at considerable risk for long-term mortality and adverse CV events [4–9]. Hence, it is critical to find potential risk factors contributing to this residual CV risk and further improve healthcare for this population.

Over the past decade, elevated levels of serum UA have not only been considered as a biomarker for gout but also an important risk factor for CVD, kidney disease, and metabolic disorders such as hypertension and diabetes [10–14]. Recently, the relationship between HUA and CV outcomes has received a large amount of attention. As reported, early onset of HUA was associated with increased CVD and mortality risk in the general population [15]. A large cohort study showed that stable high UA was correlated with an increased higher risk of MI [16]. Another study also proved a strong relationship between HUA and silent MI in healthy community dwellers [17]. A Mendelian randomization study revealed that high UA was causally related to worse outcomes, especially sudden cardiac death [18]. A meta-analysis of 14 prospective cohort studies found that for every 1 mg/dL increase in serum UA levels, the overall risk of CAD and all-cause mortality increased by 20% and 9%, respectively [19]. Apart from the general population, recent studies further confirmed an independent association of HUA with long-term CV risks in different cohorts with CAD (e.g., stable CAD, ACS, AMI, or those treated with PCI), suggesting that HUA significantly correlated with the increased CV risk [20–26]. This is not only the case in acute setting, but also in the longer term after recovery from ACS or after revascularization. Of note, some conflicting results have emerged regarding the causal effect of HUA on mortality and CV outcomes, however, the most recent studies and meta-analyses mentioned above support HUA as a residual CV risk factor.

To our knowledge, data regarding the implications of HUA in MINOCA population are scarce. A recent study enrolled 249 patients with MINOCA and reported that HUA was associated with adverse outcomes [37]. Here, we addressed this issue and conducted a larger scale and longitudinal analysis of MINOCA cohort. Consistently, we found that patients with HUA had a poorer prognosis after MINOCA. Those with HUA had a 1.49-fold higher hazard ratio of MACE compared to those without even after multivariable adjustment. HUA remained a strong predictor of MACE at PSM analysis. Further, HUA significantly improved the accuracy of risk prediction when added to the TIMI score. Apart from the composite endpoint, we found that the incidence of death, MI, stroke or revascularization did not differ significantly between HUA and non-HUA groups. There might be two reasons. First, the effect of HUA on each event may be partially attenuated by the overall improvement in clinical management of AMI and wide use of secondary prevention treatments. Second, the sample size and number of each ischemic event may be limited in this single-center study. Moreover, we found that the rate of HF was much higher in the HUA group, suggesting a role of HUA in HF progression after MINOCA. A meta-analysis also revealed that HUA increased the risk of incident HF and adverse events in HF patients [38]. Taken together, our data supported the concept that elevated UA contributes to residual CV risk and further extended the prognostic value of HUA to MINOCA patients. Given the observational design of our study, our findings should be validated by a larger randomized study confirming the causal relationship between HUA and CV outcomes after MINOCA.

The underlying mechanisms linking HUA and poor CV outcomes are manifold, and several reasons have been proposed. It is found that intracellular and mitochondrial UA aggravates atherosclerosis by releasing free radicals and inducing oxidative stress [39]. UA can also trigger inflammatory pathways, induce vascular inflammation, and further promote atherogenesis [40]. In addition, UA can penetrate endothelium, reduce synthesis and release of NO, and thus lead to endothelial dysfunction and vasoconstriction [41, 42]. Moreover, UA can promote insulin resistance and metabolic syndrome [43, 44]. All these processes may finally contribute to the development and progression of atherosclerosis, and result in a higher risk of CV events. Our study provided a rationale for identifying MINOCA patients who may benefit from UA-lowering therapies. However, the safety and long-term benefit of UA-targeted therapies in MINOCA with HUA remain unclear and warrant more research in the future.

## Limitation

Some limitations should be mentioned. First, the percentage of women was relatively low in our cohort, possibly due to the large proportion of men in all AMIs treated in our center and a lower rate for women to receive coronary angiography. Given the potential selection bias in single-center studies, future nationwide registry cohorts of MINOCA are warranted to validated our findings. Second, we did not capture and record the exact mechanism for every MINOCA patient. The association between etiology of MINOCA and outcomes should be further investigated. Third, despite multivariate adjustment and subgroup analyses were performed, there might be other unmeasured confounders that may affect the prognosis. Fourth, the serum UA levels were only measured at baseline, and the follow-up levels of UA may also be clinically significant.

## Conclusions

Hyperuricemia was an independent predictor of long-term MACE after MINOCA. In clinical practice, routine assessment of serum UA levels may help to identify high-risk patients with MINOCA and thus facilitate the pre-emptive decision making.

## Supplementary Information

**Additional file 1.** **Table S1**. Clinical risk factors in patients with or without MACE. **Table S2**. Potential clinical risk factors for MACE in MINOCA patients. **Table S3**. Distribution of clinically relevant variables and outcomes before and after propensity score matching in patients with or without hyperuricemia. **Table S4**. Uric acid and CV outcomes between sex groups. **Fig S1**. Distribution of the serum uric acid in MINOCA patients.

## Data Availability

The datasets used and analyzed during the current study available from the corresponding author on reasonable request.

## Abbreviations

ACS: Acute coronary syndrome;
AMI: Acute myocardial infarction
AUC: Areas under the curve
CAD: Coronary artery disease
CI: Confidence interval
CV: Cardiovascular
HR: Hazard ratio
HUA: Hyperuricemia
MACE: Major adverse cardiovascular event
MINOCA: Myocardial infarction with nonobstructive coronary arteries
PCI: Percutaneous coronary intervention
PSM: Propensity score matching
ROC: Receiver-operating characteristic curve
SD: Standard deviation
TIMI: Thrombolysis in myocardial infarction
UA: Uric acid
