# Supplementary material for: Hyperuricemia as a prognostic marker for long-term outcomes in patients with myocardial infarction with nonobstructive coronary arteries
Source: Nutr Metab (Lond). 2021 Dec 20;18:107. doi: 10.1186/s12986-021-00636-2 (PMC8686602; doi:10.1186/s12986-021-00636-2)
Supplement: Supplementary file 1 — Additional file 1. Table S1. Clinical risk factors in patients with or without MACE. Table S2. Potential clinical risk factors for MACE in MINOCA patients. Table S3. Distribution of clinically relevant variables and outcomes before and after propensity score matching in patients with or without hyperuricemia. Table S4. Uric acid and CV outcomes between sex groups. Fig S1. Distribution of the serum uric acid in MINOCA patients. [file 12986_2021_636_MOESM1_ESM.docx]

**Supplementary Materials of the Manuscript**

**Supplementary Table 1. Clinical risk factors in patients with or without MACE.**

|  | All MINOCA  (n=1179) | With MACE  (n=168) | Without MACE  (n=1011) | P value |
| --- | --- | --- | --- | --- |
| Male, n(%) | 867 (73.5%) | 120 (71.4%) | 747 (73.8%) | 0.504 |
| Age, yrs | 55.7±11.8 | 59.7±13.2 | 55.1±11.5 | <0.001 |
| BMI, kg/m^2^ | 25.4±3.7 | 25.4±3.8 | 25.4±3.7 | 0.924 |
| STEMI, n(%) | 475 (40.2%) | 88 (52.3%) | 387 (38.2%) | 0.001 |
| Past history, n(%) |  |  |  |  |
| Hypertension | 630 (53.4%) | 93 (55.3%) | 537 (53.1%) | 0.590 |
| Diabetes | 187 (15.9%) | 41 (24.4%) | 146 (14.4%) | 0.001 |
| Dyslipidemia | 686 (58.2%) | 105 (62.5%) | 581 (57.4%) | 0.221 |
| Previous MI | 58 (4.9%) | 9 (5.3%) | 49 (4.84%) | 0.114 |
| Killip class≥2, n(%) | 89 (7.5%) | 21 (12.5%) | 68 (6.7%) | 0.001 |
| LVEF (%) | 60.5±7.5 | 53.0±11.7 | 61.7±5.6 | <0.001 |
| Hyperuricemia, n(%) | 278 (23.5%) | 52 (30.9%) | 226 (22.3%) | 0.015 |
| Laboratory tests |  |  |  |  |
| Uric acid, μmol/L | 343.4±94.2 | 352.5±101.6 | 341.6±91.4 | 0.002 |
| HbA_1c_, % | 5.98±0.98 | 6.26±1.17 | 5.94±0.94 | <0.001 |
| Creatinine, μmol/L | 83.13±15.89 | 84.3±22.4 | 79.4±16.9 | 0.001 |
| LDL-C, mmol/L | 2.29±0.76 | 2.32±0.78 | 2.28±0.75 | 0.498 |
| hs-CRP, mg/L | 2.20 (1.03, 5.75) | 2.86 (1.95, 7.38) | 2.14 (1.02, 5.66) | 0.014 |
| NT-proBNP, pg/mL | 372 (112, 683) | 578 (214, 858) | 369 (107, 664) | <0.001 |
| Peak TnI, ng/mL | 3.24 (0.72, 6.51) | 4.32 (0.94, 8.13) | 3.13 (0.64, 6.27) | <0.001 |

Patients were divided based on the occurrence of major adverse cardiovascular events (MACE). BMI: body mass index, STEMI: ST-segment elevation myocardial infarction, LVEF: left ventricular ejection fraction, HbA_1c_: glycated hemoglobin, LDL-C: low density lipoprotein-cholesterol, hs-CRP: high-sensitive C-reactive protein, NT-proBNP: N-terminal pro-B-type natriuretic peptide, TnI: Troponin I.

**Supplementary Table 2.** **Potential clinical risk factors for MACE in MINOCA** **patients.**

| Variables | Univariate Cox analysis | | Multivariate Cox analysis | |
| --- | --- | --- | --- | --- |
|  | HR (95% CI) | P value | HR (95% CI) | P value |
| Age | 1.06 (1.02-1.11) | 0.004 | 1.02 (1.01-1.04) | 0.012 |
| Female | 1.16 (0.83-1.62) | 0.372 | NA | … |
| BMI | 0.99 (0.95-1.03) | 0.893 | NA | … |
| STEMI | 1.42 (1.05-1.92) | 0.022 | 1.22 (0.87-1.71) | 0.237 |
| Hypertension | 1.09 (0.80-1.47) | 0.575 | NA | … |
| Diabetes | 1.86 (1.31-2.65) | 0.001 | 1.50 (1.04-2.16) | 0.030 |
| Dyslipidemia | 1.18 (0.86-1.61) | 0.300 | NA | … |
| Previous MI | 1.01 (0.51-1.99) | 0.981 | NA | … |
| LVEF | 0.92 (0.91-0.93) | <0.001 | 0.96 (0.93-0.99) | 0.012 |
| ln (NT-proBNP) | 1.39 (1.22-1.58) | <0.001 | 1.17 (0.66-2.06) | 0.587 |
| Peak TnI | 1.02 (1.01-1.03) | 0.015 | 1.01 (0.99-1.02) | 0.223 |
| Creatinine | 1.04 (1.02-1.06) | <0.001 | 1.03 (1.02-1.04) | 0.035 |
| hsCRP | 1.04 (1.01-1.06) | 0.011 | 1.02 (0.99-1.04) | 0.151 |
| Hyperuricemia | 1.66 (1.18-2.32) | 0.003 | 1.59 (1.14-2.23) | 0.006 |

Statistically significant variables with univariate Cox analysis were further enrolled in the multivariate model. Hazard ratio (HR) for per 1 standard deviation increased in each continuous variable. NT-proBNP was natural logarithmically transformed to ln (NT-proBNP). NA: not assessed, CI: confidence interval, BMI: body mass index, STEMI: ST-segment elevation myocardial infarction, LVEF: left ventricular ejection fraction, NT-proBNP: N-terminal pro-B-type natriuretic peptide, TnI: Troponin I.

**Supplementary Table 3. Distribution of clinically relevant variables and outcomes before and after** **propensity score matching in patients with or without hyperuricemia.**

| Variables | Pre-PSM | | | Post-PSM | | |
| --- | --- | --- | --- | --- | --- | --- |
|  | Normouricemia  (n=901) | Hyperuricemia  (n=278) | p value | Normouricemia  (n=273) | Hyperuricemia  (n=273) | p value |
| Baseline data |  |  |  |  |  |  |
| Male, n(%) | 642 (71.2%) | 225 (80.9%) | 0.001 | 216 (79.1%) | 221 (80.9%) | 0.864 |
| Age, years | 56.6±11.1 | 53.0±13.5 | <0.001 | 53.1±12.2 | 53.1±13.4 | 0.979 |
| BMI | 25.1±3.5 | 26.5±4.3 | <0.001 | 26.1±3.8 | 26.3±4.3 | 0.738 |
| STEMI, n(%) | 344 (38.1%) | 131 (47.1%) | 0.008 | 144 (52.7%) | 145 (53.1%) | 0.932 |
| Hypertension, n(%) | 474 (52.6%) | 156 (56.1%) | 0.305 | 147 (53.8%) | 152 (55.6%) | 0.667 |
| Diabetes, n(%) | 151 (16.7%) | 36 (12.9%) | 0.129 | 39 (14.2%) | 35 (12.8%) | 0.617 |
| Dyslipidemia | 512 (56.8%) | 174 (62.5%) | 0.089 | 157 (57.5%) | 168 (61.5%) | 0.138 |
| LVEF, % | 60.8±6.7 | 60.3±8.4 | 0.112 | 60.5±7.3 | 60.2±8.5 | 0.673 |
| CV outcomes |  |  |  |  |  |  |
| MACE | 116 (12.8%) | 52 (18.7%) | 0.015 | 33 (12.1%) | 50 (18.3%) | 0.037 |
| Risk of MACE | 1 (reference) | 1.49 (1.08-2.07) | 0.016 | 1 (reference) | 1.52 (1.02-2.64) | 0.043 |

Clinically relevant variables and outcomes were compared before and after propensity score matching (PSM). The PSM was performed with a nearest neighbor approach and a 1-to-1 match between HUA and non-HUA groups. Propensity scores were calculated with a binary logistic regression model. Clinically uneven distributed variables were enrolled in PSM model, including age, sex, BMI, and MI type (NSTEMI or STEMI). Finally, 273 HUA individuals were matched to 273 non-HUA controls. Demographics and baseline comorbidities became comparable after PSM. The risk of MACE was adjusted by age, sex, BMI, MI type, hypertension, diabetes, and dyslipidemia and expressed as hazard ratio (95% confidence interval). STEMI: ST-segment elevation myocardial infarction, LVEF: left ventricular ejection fraction, MACE: major adverse cardiovascular events.

**Supplementary Table 4. Uric acid and CV outcomes between sex groups.**

|  | All MINOCA  (n=1179) | Male  (n=867) | Female  (n=312) | P value |
| --- | --- | --- | --- | --- |
| Uric acid, μmol/L | 343.4±94.2 | 363.9±89.5 | 286.6±82.8 | <0.001 |
| Presence of HUA, n(%) | 278 (23.5%) | 225 (25.9%) | 53 (16.9%) | 0.001 |
| CV outcomes, n(%) |  |  |  |  |
| MACE | 168 (14.2%) | 120 (13.8%) | 48 (15.3%) | 0.504 |
| Death, nonfatal MI, stroke  or revascularization | 102 (8.6%) | 72 (8.3%) | 30 (9.6%) | 0.480 |
| All-cause death | 18 (1.5%) | 13 (1.4%) | 5 (1.6%) | 0.899 |
| Nonfatal MI | 41 (3.4%) | 28 (3.2%) | 13 (4.1%) | 0.438 |
| Revascularization | 46 (3.9%) | 30 (3.4%) | 16 (5.1%) | 0.192 |
| Nonfatal stroke | 12 (1.0%) | 9 (1.0%) | 3 (0.9%) | 0.908 |
| Hospitalization for UA | 71 (6.0%) | 51 (5.8%) | 20 (6.4%) | 0.737 |
| Hospitalization for HF | 48 (4.0%) | 36 (4.1%) | 12 (3.8%) | 0.815 |
| Risk of MACE, unadjusted | … | 1 (reference) | 1.16 (0.83-1.62) | 0.372 |
| Risk of MACE, adjusted | … | 1 (reference) | 1.02 (0.72-1.44) | 0.916 |

Uric acid levels and CV outcomes were compared between men and women. Risk of MACE was analyzed via Cox analysis. Age, sex, BMI, MI type (STEMI or NSTEMI), hypertension, diabetes and dyslipidemia were included in multivariate Cox analysis. HUA: hyperuricemia, MACE: major adverse cardiovascular events, UA: unstable angina, HF: heart failure.

**
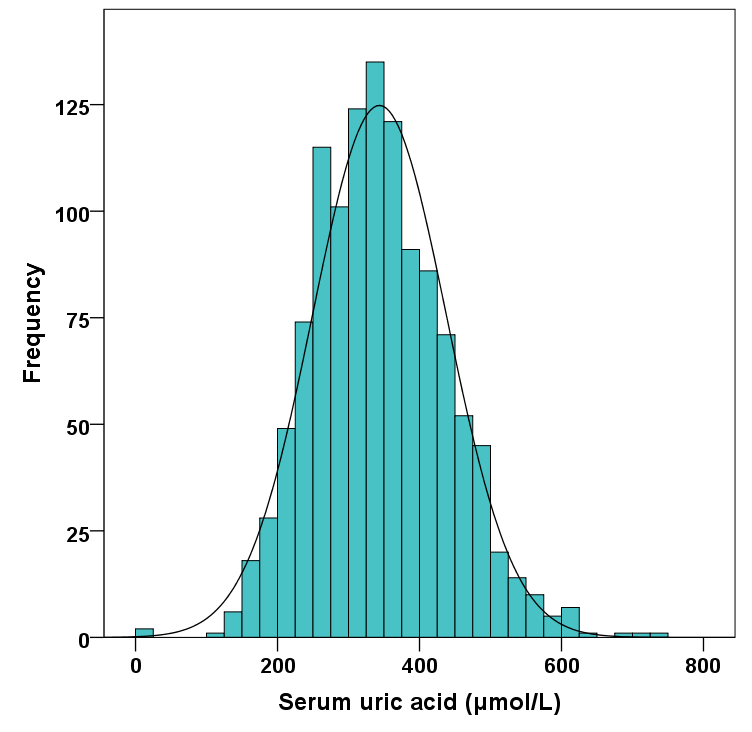
**

**Supplementary Figure 1. Distribution of the serum uric acid in MINOCA patients.**
